# Strategies for Developing an Inclusive Geriatric Physician Workforce for Older Adults in the U.S

**DOI:** 10.1093/geroni/igab046.2819

**Published:** 2021-12-17

**Authors:** Brandy Wallace, Leanne Clark-Shirley, Pallavi Podapati

**Affiliations:** 1 University of Maryland, Baltimore County, Baltimore, Maryland, United States; 2 American Society on Aging, American Society on Aging, California, United States; 3 Princeton University, Princeton University, New Jersey, United States

## Abstract

The “geriatric imperative” has been part of the aging discourse for more than 30 years but neither geriatric practice nor older adults are homogenous. As the U.S. population ages, elders will become more racially and ethnically diverse; and, their health outcomes will be shaped by lifetime experiences with systemic discrimination and racism. Already, COVID-19 has made clear that older adults and non-Whites, particularly African Americans and Hispanics, disproportionately bear the burden of disease and illness. Research suggests health disparities will continue unless there is change within the health care system. The Institute of Medicine (2001) reported on the problematic nature of the stark contrast between the diversity of patients and the physicians caring for them, including issues with patient trust and communication, yet no significant movement has been made to diversify the physician workforce. Despite being 13% and 16% of the U.S. population, respectively, African Americans and Hispanics make up just 5% and 6% of the practicing physician workforce. Further, practicing geriatricians represent less than 1% of physicians with very few physicians of color. There is a need for more African American and Hispanic geriatricians. In this systematic review, we examine recruitment and retention efforts targeting students of color, and curricula of geriatric medical programs in the U.S. We offer recommendations toward incentivizing physicians of color to enter geriatrics, strategies to support decolonization of geriatric medical curricula in undergraduate medical education programs, and the development of mentorship and pipeline programs to increase diversity in the geriatric physician workforce.

